# Cascade-Enhanced Lateral Flow Immunoassay for Sensitive Detection of Okadaic Acid in Seawater, Fish, and Seafood

**DOI:** 10.3390/foods11121691

**Published:** 2022-06-09

**Authors:** Olga D. Hendrickson, Elena A. Zvereva, Anatoly V. Zherdev, Boris B. Dzantiev

**Affiliations:** Bach Institute of Biochemistry, Research Center of Biotechnology of the Russian Academy of Sciences, Leninsky Prospect 33, 119071 Moscow, Russia; odhendrick@gmail.com (O.D.H.); zverevaea@yandex.ru (E.A.Z.); zherdev@inbi.ras.ru (A.V.Z.)

**Keywords:** phycotoxins, okadaic acid, lateral flow immunoassay, signal amplification, seawater, seafood

## Abstract

In this investigation, a new approach for developing a sensitive lateral flow immunoassay (LFIA) was proposed for the detection of the hazardous marine toxin okadaic acid (OA). It is based on the indirect format with anti-species antibodies labeled by gold nanoparticles (AuNPs) and cascade signal amplification. The latter is performed by first passing a mixture of anti-OA antibodies and a tested sample along the immunochromatographic test strip and then performing several cycles of the interaction of anti-species antibodies conjugated with AuNPs with free antibodies, which bind to anti-species antibodies but are not specific to the target analyte. As a result, branched aggregates are formed, due to which the colorimetric signal intensification occurs. The developed test system enabled the detection of OA with an instrumental detection limit of 30 pg/mL and a cutoff of 1 ng/mL, which exceeds these characteristics in the LFIA without amplification by 7 and 2 times, respectively. The OA recoveries from seawater, fish, and seafood varied from 76.9% to 126%. The test system may be required for point-of-care monitoring of samples for phycotoxin contamination; the developed principle of signal amplification can be used in cases where highly sensitive detection of trace amounts of a contaminant is required.

## 1. Introduction

Among a large number of compounds related to food contaminants, a special place belongs to phycotoxins—extremely toxic compounds produced by microalgae and cyanobacteria that are part of the plankton and benthos of the world ocean [1,2,3]. Normally, algae and cyanobacteria are a necessary component of aquatic ecosystems; however, under favorable conditions, these organisms actively multiply, which leads to the so-called water bloom (for example, “red tides”) and changes in ecosystems [4]. Algae and cyanobacteria are an intermediate link in the food chain, serving as food for macroorganisms such as fish and shellfish. The transfer of phycotoxins to aquatic animals can lead to significant environmental and economic consequences causing their death and thereby harming fish farming and fisheries [5]. In addition, water containing phycotoxins is not suitable for drinking because most of them are thermostable, which causes difficulties for purification in the water supply. Human consumption of fish and shellfish contaminated with phycotoxins leads to poisoning, sometimes massive because phycotoxins affect health even in small concentrations [6,7].

Phycotoxins produced by several species of dinoflagellates and causing diarrheic shellfish poisoning (DSP) include, in particular, okadaic acid (OA), the mechanism of action of which is manifested in the inhibition of protein phosphatase activity [8,9]. Because OA is a lipophilic compound, it accumulates in the fatty tissues of shellfish and fish [9]. DSP, which develops almost immediately after the intake of OA-containing seafood, is characterized by nausea, vomiting, abdominal pain, and profuse diarrhea [10]. Given the high toxicity of OA, its content in food products is strictly regulated. Thus, according to the European Union regulatory limit, the content of OA in mollusk tissues should not exceed 0.16 µg/kg [11].

Ensuring food quality and safety requires the control of the contamination of raw materials, semi-finished, and finished food products. This requirement applies in particular to fish, seafood, and related foodstuffs, which due to their palatability and nutritional value are included in the diet in many countries and are the basis for standard and gourmet dishes. Therefore, phycotoxins are included in the list of mandatory controlled food contaminants, and analytical methods for their detection are an essential tool to implement their monitoring. For precise and sensitive determination of phycotoxins, complex analytical methods such as high-performance liquid chromatography–mass spectrometry are often used, which require specialized laboratories with complex and expensive equipment and highly qualified operators [12,13]. These approaches cannot provide a rapid point-of-care determination of the toxicant, especially for mass screening of samples. From this point of view, immunochemical methods, particularly the LFIA based on a combination of chromatography and highly specific interaction of analytes with antibodies, can be an alternative or addition to complex arbitrage analytical methods. It provides rapid results (10–20 min) on not only qualitative (phycotoxin presence/absence) but in many cases also quantitative (its concentration) characteristics [14,15,16].

It should be noted that the peculiarity of multicomponent food and water matrices is that before analysis, a sample preparation procedure often associated with multiple dilutions of samples is required. As a result, the sensitivity of the analysis developed in model conditions (determination in a buffer) can be insufficient to ensure a reliable detection in real phycotoxin-containing samples. Therefore, it is necessary to have a margin in the assay sensitivity that allows for the reliable detection of a phycotoxin in the sample. Therefore, the creation of approaches aimed at lowering the limit of detection (LOD) is an extremely popular direction in the development of analytical systems including LFIAs [17,18,19,20].

The LFIA of OA has been described in several studies [21,22,23,24,25]. Most of these works are based on the routine direct competitive LFIA with AuNPs as a label for specific antibodies. The reported test systems enable the determination of OA with LODs varying in the range of 0.1–50 ng/mL. Only one recent study is devoted to the development of an enhancement strategy based on the catalysis of Au@Pt nanoparticles and horseradish peroxidase [25]. With this approach, the authors achieved an OA LOD of 0.04 ng/mL. The developed LFIAs were tested for the detection of OA in real samples of shellfish.

In contrast to the studies described above, where the same principle of competitive interaction and one-stage assembling of a detectable complex are reported, in this study, a highly sensitive indirect LFIA of OA was developed based on the amplification of the analytical signal, which is provided by a cascade of interactions between gold-labeled secondary antibodies and free antibodies having specificity to these secondary antibodies but not to OA. The achieved analytical characteristics exceed those in all published studies on OA immunochromatography, including an enhanced LFIA. The developed enhanced LFIA was applied for the detection of OA in spiked samples of seawater, fish, and seafood.

## 2. Materials and Methods

### 2.1. Reagents, Materials, Equipment, and Software

OA, gold (III) chloride hydrate (HAuCl_4_ × H_2_O), sodium azide, methanol, sucrose, Triton X-100, and bovine serum albumin (BSA) (Sigma-Aldrich, Saint Louis, MO, USA) were used. Goat anti-mouse immunoglobulins (GAMI) and donkey anti-goat immunoglobulins (DAGI) were purchased from Arista Biologicals (Allentown, PA, USA). Monoclonal antibodies (MAbs) to OA (clone 7E1) were purchased from Santa Cruz Biotechnology (Dallas, TX, USA). All other compounds were analytically pure.

For the LFIA, a CNPC-SS12 nitrocellulose membrane fixed on the plastic support and a GFB-R4 membrane (Advanced Microdevices, Ambala Cantt, India) were used as a working membrane and a sample pad, respectively. As an adsorption pad, a ReliaFlow 319 membrane (Ahlstrom-Munksjö, Helsinki, Finland) was applied.

Transmission electron microscopy (TEM) was performed on a CX-100 microscope (Jeol, Tokyo, Japan). A Zenyth 3100 vertical photometer (Anthos Labtec Instruments, Wals, Austria) was used to register the optical density (OD) of gold solutions. An Iso-Flow dispenser (Imagene Technology, Hanover, NH, USA) was utilized to apply the reagents on the immunochromatographic working membrane (at a rate of 0.1 μL per mm), and an automatic guillotine (KinBio, Shanghai, China) was used to cut it into test strips. To assess bands’ coloration, a CanoScan LiDE 90 scanner (Canon, Tokyo, Japan) and TotalLab software (Nonlinear Dynamics, Newcastle upon Tyne, Great Britain) were used. Origin software (OriginLab, Northampton, MA, USA) was applied to estimate the analytical characteristics of the developed test systems.

### 2.2. Synthesis of AuNPs and Their Conjugation with GAMI

AuNPs were obtained by the standard approach described in [26] and characterized by TEM as reported in [27]. To determine the GAMI concentration for conjugation with AuNPs, a flocculation curve was obtained. The pH of the AuNP solution (OD_520_ = 1) was adjusted to 9.0 with 100 mM sodium carbonate. After that, AuNPs (500 μL) were mixed with GAMI solutions (0.5–200 μg/mL, 50 μL in 10 mM Tris-HCl, pH 8.5) and incubated for 10 min at room temperature. Then, 10% sodium chloride (50 μL) was added and OD_580_ was measured after stirring. Finally, the dependence of OD_580_ versus GAMI concentration was built. OD_580_ was chosen because of the changes in spectral characteristics of AuNPs solution after the aggregation of nanoparticles caused by the addition of the coagulating agent (NaCl) [28].

To obtain labeled antibodies, GAMI were added to AuNPs (OD_520_ = 1, pH 9.0) in the concentration of 6 μg/mL. The mixture was shaken for 45 min at room temperature, followed by the addition of a 10% water solution of BSA (40:1, *v*/*v*) and vigorous stirring for 15 min. Then, the GAMI–AuNPs conjugate was pelleted by centrifugation at 9500× *g* for 35 min at 4 °C. The precipitate was resuspended to an OD_520_ = 15 in 10 mM Tris buffer, pH 8.5, containing 1% BSA, 1% sucrose, and 0.1% sodium azide. The conjugate was stored at 4 °C.

### 2.3. Preparation of Test Strips

In the case of the standard LFIA, test strips were combined from the working membrane, a sample pad, and an adsorption pad. For the enhanced LFIA, the plastic support was cut until the lower edge of the working membrane. For both formats of the LFIA, OA–BSA (0.5 mg/mL in PBS) and DAGI (0.1 mg/mL in PBS for the standard LFIA and 0.05 mg/mL for the enhanced LFIA) were applied onto a working membrane to form a test (T) zone and a control (C) zone, respectively. The multimembrane composite was dried overnight at room temperature and for 1.5 h at 37 °C and then cut into strips of 3.0 mm width. The strips were stored at room temperature in a sealed package with silica gel.

### 2.4. Pretreatment of Seawater and Seafood Samples

A seawater sample was taken from the Aegean Sea (Fethiye region, Turkey) and stored at 4 °C. Before analysis, Triton X-100 was added to seawater (0.05%). Then, the obtained mixture was diluted by 10 times with PBST and spiked with known concentrations of OA.

The real seafood samples included fish (trout from the Barents Sea, Russia), tiger shrimps, and scallops (both from the Sea of Okhotsk, Russia) purchased from local food stores. For the sample preparation of fish and seafood, the following technique was used: first, samples were minced into a homogeneous mass using a household blender. Then, to a 0.5 g sample, OA (50 μL, 1 μg/mL, which corresponds to 100 ng/g), and 5 mL of the methanol–water mixture (1:1) were added. The mixtures were stirred for 5 min and centrifuged at 1500× *g* for 10 min. The supernatants were collected and stored at −18 °C. Before the LFIA, the extracts were diluted 10 times with PBST.

### 2.5. LFIA of OA

For the determination of OA, its solutions (15.2 pg/mL–100 ng/mL, 50 μL in PBST) were mixed with anti-OA MAbs (0.1 μg/mL, 50 μL in PBST) and GAMI–AuNPs (2.5 μL, OD_520_ = 15) and incubated for 3 min at room temperature. Then, the test strips were incubated in the obtained solutions for 15 min. To estimate the LFIA results, test strips were removed from the solutions, blotted and scanned. Then, bands’ coloration in the T zone was assessed.

### 2.6. LFIA of OA with Cascade Signal Amplification

OA solutions (0.08 pg/mL–50 ng/mL, 10 μL in PBST) were mixed with anti-OA MAbs (0.01 μg/mL, 10 μL in PBST) and incubated for 3 min at room temperature. Then, the test strips were immersed into the mixture and incubated for 5 min. After that, the test strips were transferred to the solution of GAMI–AuNPs (2 μL in 20 μL of PBST) and incubated in it. This and all other stages were carried out for 7 min. Then, 2 cycles of signal amplification were performed. A single cycle included the following steps: the test strips were transferred to the solution of DAGI (20 μL, 500 ng/mL in PBST) and, after incubation, were transferred to the solution of GAMI–AuNPs (2 μL in 20 μL of PBST). Finally, the test strips were processed as described above.

In the case of the enhanced LFIA in real samples, spiked extracts of fish or seafood or seawater pretreated as described above were added instead of OA buffer solutions. All other stages of the analysis were the same.

### 2.7. Evaluation of the Assay Results

The plots of color intensity or OD (y) versus the OA concentrations (x) were built and fitted to a four-parameter logistic function using Origin software (OriginLab, Northampton, MA, USA). The LODs, cutoffs, and working ranges were evaluated as described in [28,29].

## 3. Results and Discussion

### 3.1. Obtaining the Immunoreagents

**Figure 1:**
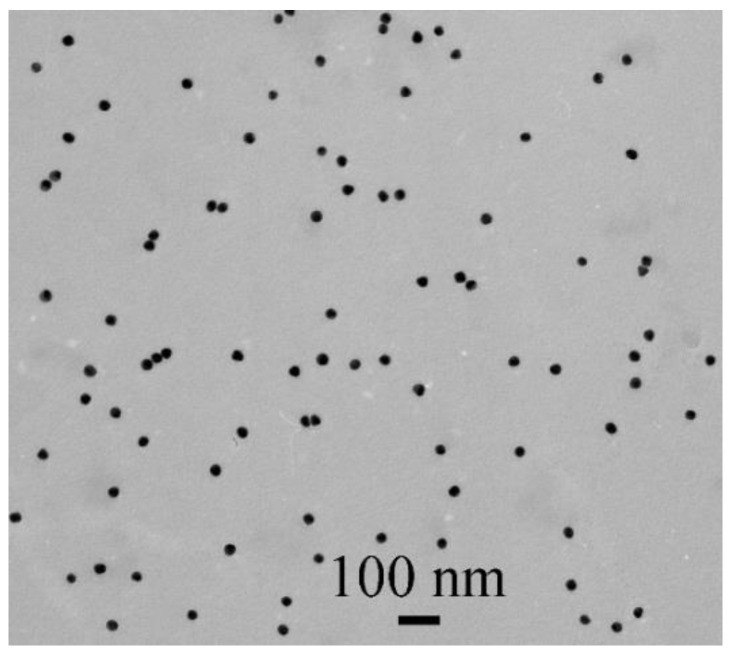
TEM microphotograph of AuNPs.

To develop the LFIA of OA, colloidal gold was used as a traditional label in immunochromatography, which is characterized by a standardized synthetic protocol, long-term stability, and a high colorimetric signal that provides sensitive and reliable analyte determination both visually and instrumentally. AuNPs were obtained through the reduction of HAuCl_4_ with sodium citrate [26]. In this study, AuNPs with a diameter of about 30 nm were synthesized as the most optimal marker in the LFIA [30]. TEM characterization showed that the sample contained homogeneous non-aggregated particles. The average diameter of AuNPs (a sample containing 201 nanoparticles was processed) was 30.9 ± 3.4 nm with a minimum value of 23.5 nm and a maximum value of 39.2 nm; the ellipticity was 1.1 ± 0.06 (Figure 1).

**Figure 2:**
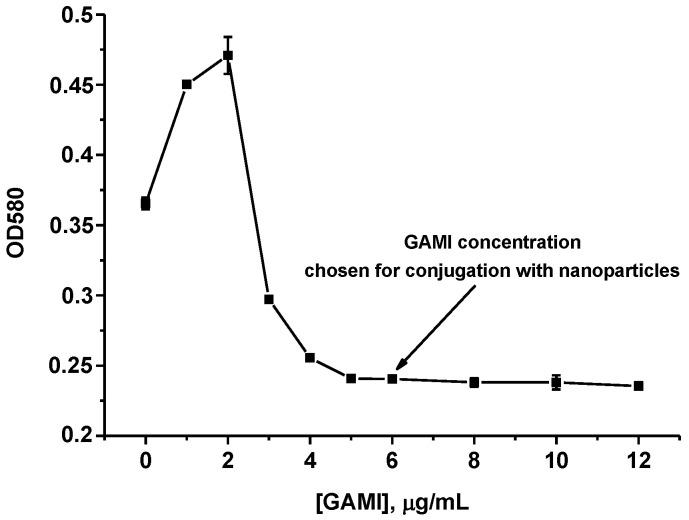
GAMI flocculation curve.

Both the standard and enhanced LFIAs were implemented in an indirect format implying conjugation with a label of not specific (anti-OA MAbs), but anti-species antibodies (GAMI). Before obtaining the GAMI–AuNPs conjugate, it was necessary to determine the concentration of antibodies used for complexation. This stage is very important because a correctly determined quantitative ratio of the marker and antibodies ensures the stability of the immunocomplex in media with different pH and ionic strength. The choice of GAMI concentration was carried out with the help of a flocculation curve—the dependence of the OD of the colloidal gold solution on the concentration of added antibodies in a medium with a high content of a coagulator (10% NaCl). At an insufficient concentration of antibodies, AuNPs have an unstabilized surface and are likely to aggregate, which is visualized as a change in the shade of the colloidal gold solution towards violet (a growth of the OD on the flocculation curve is observed, Figure 2).

With an increase in protein concentration, the OD decreases reaching a plateau (a flocculation point), which indicated that the surface of AuNPs becomes steady and their aggregation stops. An antibody concentration corresponding to (or slightly above) the flocculation point is usually used to obtain a stable GAMI–AuNPs complex [27]. In our case, it corresponded to 6 μg of GAMI per 1 mL of AuNPs solution (indicated by an arrow in Figure 2).

Commercial MAbs were used as a receptor for OA. To confirm their reactivity toward OA, primary characterization (without optimization) was carried out by the indirect enzyme-linked immunosorbent analysis (ELISA), which showed that the LOD of OA was 0.5 ng/mL (Figure S1).

### 3.2. Standard LFIA of OA

As it was noted above, the standard LFIA was performed in the indirect format based on the competition between free OA in the sample and its protein conjugate immobilized on the immunochromatographic membrane for the binding to anti-OA MAbs. Red-colored GAMI–AuNPs were used to reveal the immune complexes formed in the test strip. A tested sample was mixed with OA-specific antibodies and GAMI–AuNPs and incubated for a short time before dipping the test strip. Then, test strips with immobilized OA–BSA in the T zone and secondary antibodies specific to GAMI in the labeled conjugate (DAGI) adsorbed in the C zone were incubated with the reaction mixture. In the presence of OA, the latter blocks specific antibodies averting the formation of the OA–BSA–MAbs–GAMI–AuNPs complex in the T zone and thereby preventing the appearance of the colored band. Contrariwise, in the absence of OA in the sample, MAbs bound with GAMI–AuNPs interact with the immobilized antigen causing the formation of a colored band. In the C zone, coloration occurs in any case owing to interaction of an excess of the labeled conjugate with DAGI adsorbed there. Thus, the intensity of the coloration in the T zone is measured to assess the concentration of OA in the sample.

For the correct comparison of the standard and enhanced variants of the LFIA, it was necessary to optimize both formats in terms of achieving the lowest possible LODs while maintaining analytical signal amplitudes sufficient for reliable determination. This was implemented by varying the assay conditions—the duration of its stages and the reagents’ concentrations (Table S1). As a result, it was found that this demand was fulfilled if specific antibodies were added at a concentration of 0.1 µg/mL (at a lower amount, the intensity of the analytical signal decreased and did not meet the requirement for the assay accuracy; at a higher concentration, the LOD undesirably increased). The optimal volume of the GAMI–AuNPs conjugate added to the sample was 2.5 μL. With a smaller amount of the marker, the coloration of the zones was too pale, that is, the signal amplitude decreased; a larger amount slightly increased the brightness of the zones but the background signal and the consumption of the reagent also increased. The 3-min duration of the preincubation stage was chosen as sufficient for the progress of homogeneous immune reactions. The incubation of the test strip with the sample was carried out for 15 min, the time sufficient for the lateral flow of the reaction mixture along the membrane carriers and the implementation of all required interactions.

**Figure 3:**
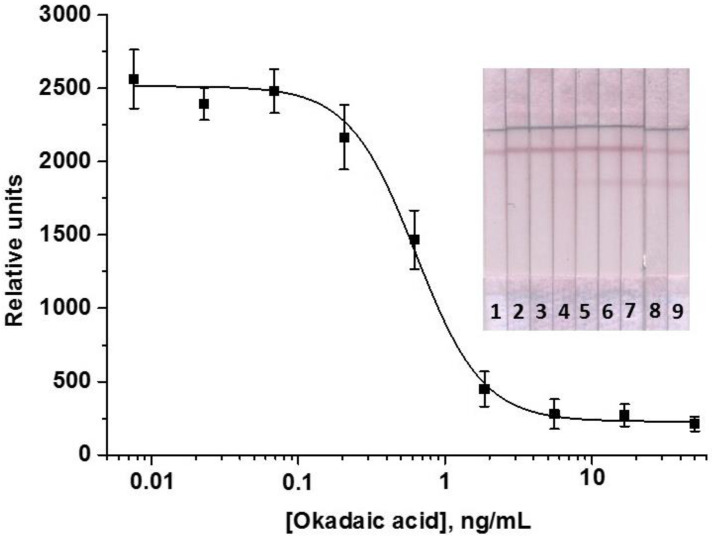
Calibration curve of OA in the LFIA (*n* = 3) and the corresponding test strips. Concentrations of OA were 50 ng/mL (1); 16.7 ng/mL (2); 5.6 ng/mL (3); 1.9 ng/mL (4); 0.62 ng/mL (5); 0.21 ng/mL (6); 69 ng/mL (7); 23 pg/mL (8); 7.6 pg/mL (9).

The LFIA optimization allowed achieving high analytical parameters: the instrumental LOD of OA was 0.2 ng/mL and the working range of the detectable concentrations was 0.31–1.3 ng/mL. Visual LOD (cutoff) was 2 ng/mL. The OA calibration curve and the test strips corresponding to concentrations plotted on the curve are shown in Figure 3. According to the obtained data, the signal amplitude reached about 2500 relative units (RU).

### 3.3. LFIA of OA with Cascade Signal Amplification

The proposed method of signal amplification is performed through a cascade of immunochemical reactions occurring on the test strip, which leads to the progressive increase in the intensity of zones’ coloration [31] (see the scheme of the enhanced LFIA in Figure 3). It consists of passing a solution of specific MAbs mixed with an antigen-containing sample along the test strip followed by several (at least two) cycles of successive passing gold-labeled anti-species antibodies (GAMI–AuNPs) and free anti-GAMI antibodies that are not specific to OA (DAGI). The result of these processes is the formation of aggregates with a (GAMI–AuNPs − DAGI) × *n* structure, where *n* is the number of GAMI–AuNPs/DAGI passing cycles. Theoretically, the number of such cycles (and, accordingly, the number of layers formed in the T zone) is unlimited, which eliminates stoichiometric restrictions on the amount of the markers attached to one immunoreagent molecule immobilized on the membrane.

**Figure 4:**
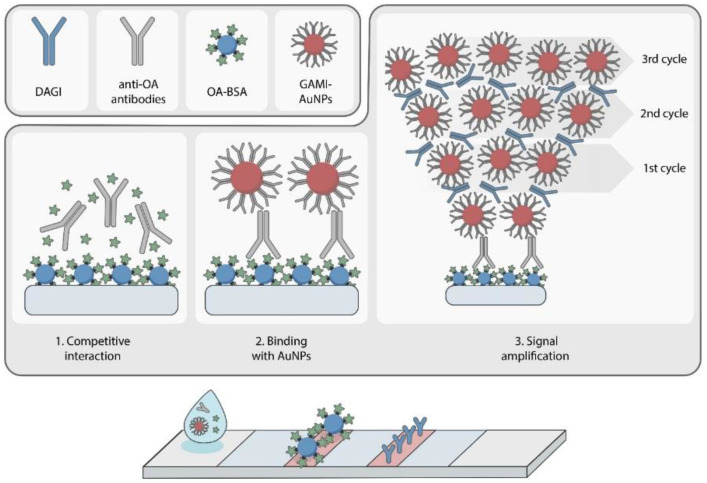
Scheme of the LFIA with cascade signal amplification.

Under cascade amplification, the stages of specific interaction with the antigen in the sample and the introduction of a colored label into the detected complex were separated. During the first reaction, the MAbs–OA–BSA complex is formed in the analytical zone. For its detection, a second reaction is performed: a solution of GAMI–AuNPs is passed along the test strip, which leads to their binding in the T zone and the appearance of a colored band. It should be noted that the AuNPs are evenly coated with anti-species antibodies. Hence, if binding with the MAbs–OA–BSA complex occurs on one side of the nanoparticle, the opposite side remains free for other interactions. Therefore, it is possible to carry out additional interactions that increase the incorporation of the marker into the immune complex. For this purpose, a solution of free antibodies (DAGI, not specific to the target analyte but binding to anti-species antibodies that are already included in the complex on the membrane) is passed along the test strip. Then, a solution of GAMI–AuNPs is again passed to label the resulting new layer of immunoglobulins. As a result, a complex multilayer structure is formed in the T zone, where one antigen–antibody complex induces the binding of a large amount of colored marker (Figure 4). The separation of stages enables independent control of the content of specific antibodies and a colored marker in the system, which, in turn, allows for increasing the color intensity of the T zone and thereby reducing the LOD of the analyte.

When developing the enhanced LFIA, multifactorial optimization was carried out because such a format differed significantly from the standard one and required the selection of many assay parameters. First, it was necessary to establish the number of amplification cycles that have to be included in the analysis taking into account that the aim of the development is the maximum decrease in the LOD of OA. According to the principle described above, it is achieved by increasing the number of amplification cycles. However, a large number of the assay stages will prolong its total duration. Regarding this, it was necessary to choose such detection conditions under which the achievement of high analytical parameters of the test system does not contradict the rapid detection as the basic advantage of the LFIA.

The enhanced LFIA of OA was initially carried out under the same conditions as the standard one, i.e., with the same composition of test strips, the volume of the analyzed sample (100 μL), the concentration of reagents deposited on the working membrane, specific antibodies (0.1 μg/mL), and GAMI–AuNPs in solution (2.5 μL for each cycle). Under these conditions, one or more amplification cycles were implemented by sequentially as test strips were incubated in GAMI–AuNPs and DAGI solutions. As a result of the experiments, several important features were revealed.

First, at least 12–15 min duration of the incubation of full-size test strips with a 100 μL reaction mixture is required to ensure maximum liquid absorption and effectiveness of immune interactions on the membrane. Consequently, the detection with 1–3 amplification cycles takes approximately 50–100 min, which is comparable to the microplate ELISA and seriously contravenes the LFIA rapidity. The total time for the liquid movement along the test strip consists of the durations of movements along its components, including a sample pad. Therefore, we decided to cut the test strips up to the lower edge of the working membrane, thus reducing the length of the strip by about one-third. In this case, the sample volume of 100 μL also became excessive and was reduced. As a result of the experiments on the selection of the combination of the volume of the reaction mixture and the optimal time of its incubation with the test strip, the 20 μL sample volume (we varied it in the range of 15–30 μL) and 7 min incubation time (we varied in the range of 5–10 min) were chosen. For the initial incubation of a dry test strip with OA and MAbs-containing sample, 5 min was sufficient to completely absorb a reaction mixture. Therefore, the total assay duration was 29 min for one cycle and increased by 14 min for each subsequent cycle. In this case, it was irrational to carry out more than 2–3 amplification cycles from the point of view of rapid detection.

**Figure 5:**
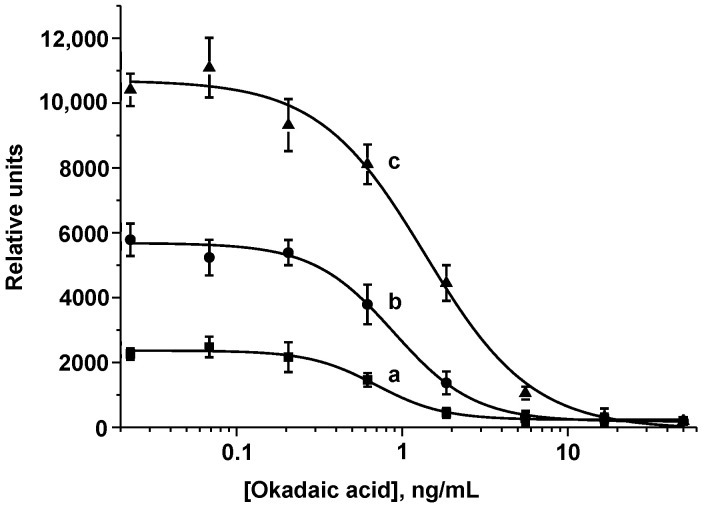
Calibration curves of OA in the standard LFIA (**a**) and in the enhanced LFIA with 2 (**b**) and 3 (**c**) amplification cycles (*n* = 3).

Secondly, when using the same concentration of anti-OA MAbs as in the standard LFIA, the intensity of the colorimetric signal in the enhanced LFIA significantly increased (up to >10,000 RU) depending on the number of amplification cycles (Figure 5).

For a correct comparison with the standard LFIA, it was advisable to unify the colorimetric signal generated as a result of detection (~2500 RU). It was achieved by reducing the concentration of specific antibodies, which, in turn, decreased the OA LOD. In the enhanced LFIA, the concentration of MAbs was reduced by an order of magnitude (to 10 ng/mL, we varied it in the range from 8 to 40 ng/mL). It should be noted that under this concentration of MAbs, the coloration in the T zone is absent in the standard LFIA. At the same time, because the number of incubations with GAMI–AuNPs increases by at least one (with a single amplification cycle) and at each stage the coloration in the zones becomes brighter, it becomes possible to reduce the volume of the added GAMI–AuNPs conjugate. Finally, 2 μL of the GAMI–AuNPs conjugate was added at each amplification stage. On the one hand, this is slightly less than the volume added in the standard LFIA, but on the other hand, due to a significant decrease in the concentration of anti-OA MAbs, the increase in the signal occurs gradually precisely during the cascade amplification. Another parameter for optimization was the DAGI concentration. The concentration of 500 ng/mL was chosen as optimal (we varied it in the range of 150–1000 ng/mL). At a lower amount of DAGIs, the enhancement of zones’ coloration during the cascade was not sufficiently pronounced; at a higher amount, the background signal increased significantly. Because during cascade amplification, an increase in the signal also occurred in the C zone of the test strip, the DAGI concentration there was 2 times reduced compared to the standard LFIA (to 0.05 ng/mL). The concentration of the OA–BSA conjugate in the T zone was unchanged (0.5 ng/mL).

**Figure 6:**
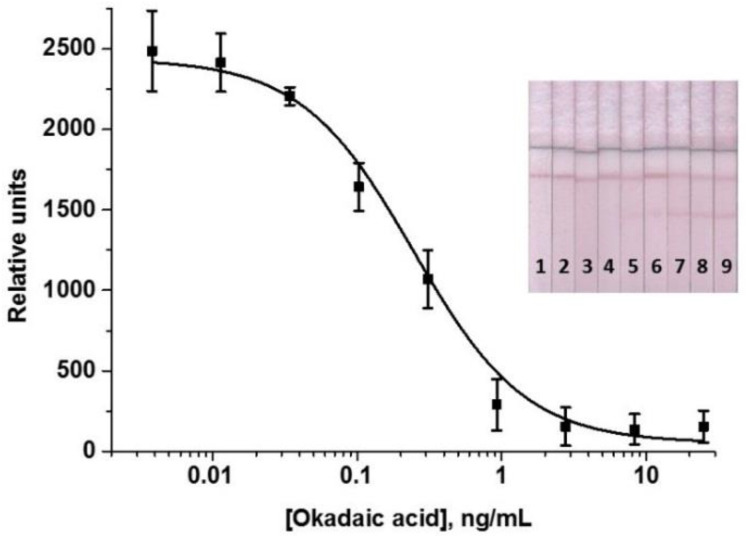
Calibration curve of OA in the enhanced LFIA (*n* = 3) and the corresponding test strips. Concentrations of OA were 25 ng/mL (1); 8.3 ng/mL (2); 2.8 ng/mL (3); 0.93 ng/mL (4); 0.31 ng/mL (5); 0.10 ng/mL (6); 34 pg/mL (7); 1.2 pg/mL (8); 0.04 pg/mL (9).

Under the conditions chosen during optimization, the LFIAs with one, two, and three amplification cycles were implemented. With a single amplification cycle, the amplitude of the analytical signal was too low (<2000 RU), which negatively affected the reliability and accuracy of the analysis. Therefore, this variant was excluded from the comparison, and only two- and three-cascade formats were considered. It was demonstrated that at comparable instrumental LODs, the visual LOD (cutoff) after three amplifications increased. This can be explained by the growth of the background signal as a result of non-specific interactions that may occur as a result of a significant increase in the incubation time of the test strip during multiple steps of the three-stage amplification. In addition, the total assay duration for three cascades was 57 min. Thus, the LFIA with two cycles of signal amplification was chosen. The OA calibration curve obtained in the optimized LFIA with cascade amplification is shown in Figure 6. The OA LOD was 0.03 ng/mL, which is almost 7 times lower than in the standard LFIA; the working range of the detectable concentrations was 0.07–0.85 ng/mL. Cutoff decreased by 2 times—down to 1 ng/mL. The obtained results allowed for testing the developed analysis for detecting OA in real samples.

### 3.4. Enhanced LFIA of OA in Seawater, Fish, and Seafood

As real samples, natural seawater as well as samples of fish (trout) and seafood—tiger shrimps (as a representative of crustaceans) and scallops (as a representative of mollusks)—were used. In this study, samples of sea trout were used to develop a method for sample preparation and further LFIA. Freshwater trout can also be contaminated with DSP toxins produced by dinoflagellates, which are the important group of phytoplankton in marine and fresh waters [32]. Rapid and simple sample preparation techniques were proposed. Triton X-100 detergent was added to seawater to increase the mobility of the reaction mixture along the immunochromatographic membrane and decrease the non-specific binding. Then, seawater was diluted with PBST. For fish and seafood, methanol:water extraction was applied, followed by the dilution of the extracts with PBST.

Using it, both blank and spiked samples were processed. In the latter case, sample preparation was aimed not only at reducing the matrix effect on the results of testing, but also at effective OA extraction. Confirmation of the OA absence in seawater, fish, and seafood (before spiking) was carried out using OA ELISA kits (EuroProxima, Arnhem, the Netherlands). OA recovery values calculated on the base of the OA calibration curve in PBST are presented in Table 1.

The data in Table 1 indicate that the developed LFIA enables the determination of 76.9–126% OA in seawater, fish, and seafood samples. The accuracy of the developed assay was confirmed using the OA ELISA kits (EuroProxima, Arnhem, the Netherlands). High correlation coefficients (0.985, *n* = 10) between the amounts of OA determined by the LFIA and the ELISA were demonstrated.

### 3.5. Advantages of the Developed Test System

Comparison with previously reported studies on the LFIA of OA demonstrates that the achieved LOD (30 pg/mL) and cutoff (1 ng/mL) are the minimum among all reported studies even that where amplification approach is used (Table 2). The assay sensitivity is much beyond the official requirements, which allows varying sample preparation conditions, including significant dilution of complex matrix samples. In addition, the proposed amplification approach is very simple and does not require the synthesis of any new immunoreagents and labeled complexes: the enhanced LFIA includes the same set of reagents as the conventional indirect competitive LFIA. As additional advantages of the developed test system, a very small volume of the test sample (20 μL) and extremely low consumption of specific antibodies may be noted.

Unlike previous works, in our investigation, the analysis of real matrices was not limited only to seafood. We enlarged a panel of tested samples to seawater as a primary target for contamination of phycotoxins and representatives of fish (trout), mollusks (scallops), and crustaceans (tiger shrimps), which additionally confirmed the competitive capabilities of the test system. The sample processing procedure was very simple and, most importantly, short: sample preparation of fish and seafood took only 20 min, and for seawater, it was reduced to a simple dilution with a buffer.

## 4. Conclusions

An approach for a very sensitive determination of OA as a hazardous marine toxin was developed. It was based on the indirect lateral flow immunoassay with signal amplification carried out due to the introduction of a large amount of marker into the test system. Several successive cycles (a cascade) of the interaction with gold-labeled anti-species antibodies ensure the formation of branched structures, thus significantly increasing the amount of the marker attached to the initial immune complex. The LOD, cutoff, and linear range of the test system were 0.03, 1, and 0.07–0.85 ng/mL, respectively. The LFIA was successfully applied to detect OA in spiked samples of seawater, fish, shrimps, and scallops with the recoveries of 76.9% to 126%, which confirms the promise of this method for the sensitive detection of phycotoxins in various objects. A comparison of the developed cascade-enhanced lateral flow immunoassay with already used methods demonstrated that this method required less time and successfully detected OA.

## Figures and Tables

**Table 1:** Recoveries of OA from seawater, fish, and seafood (*n* = 3).

Seawater						
Added OA, ng/mL		Detected OA ± SD ^1^ (ng/mL)		Recovery ± SD (%)		
0.5		0.45 ± 0.04		89.2 ± 8.1		
0.75		0.62 ± 0.04		82.0 ± 4.7		
**Fish and Seafood**						
Added OA, ng/mL	Detected OA ± SD (ng/g)/	Recovery ± SD (%)	Detected OA ± SD (ng/g)/	Recovery ± SD (%)	Detected OA ± SD (ng/g)/	Recovery ± SD (%)
	Trout		Shrimps		Scallops	
50	38.5 ± 1.0	76.9 ± 1.9	47.8 ± 5.7	95.60 ± 11.4	51.9 ± 2.1	103.7 ± 4.2
100	113.9 ± 10	113.9 ± 10	123 ± 1.2	123 ± 1.2	126 ± 15	126 ± 15

^1^ SD—standard deviation, *n* = 3.

**Table 2:** Comparison of the developed LFIA with previous studies in this field.

LFIA Format	Label	LOD, ng/mL	Cutoff, ng/mL	Detected Real Samples	Reference
Direct competitive	Anti-OA MAbs—AuNPs	10	50	Shellfish	[23]
Direct competitive	Anti-OA MAbs—AuNPs	3.12	6.25	Mussels	[20]
Direct competitive	Anti-OA MAbs—AuNPs	n/p ^1^	5	Clams, scallops, mussels, and oysters	[22]
Direct competitive	Anti-OA MAbs—AuNPs	100	1	Shellfish	[21]
Catalysis enhancement	Anti-OA MAbs—Au@PtNPs	0.04	n/p	Oysters, mussels, and clams	[24]
Indirect competitive	GAMI–AuNPs	0.03	1	Seawater, fish (trout), tiger shrimps, and scallops	This study

^1^ Not presented.

## Data Availability

The original contributions presented in the study are included in the article and Supplementary Materials; further inquiries can be directed to the corresponding author.

## Supplementary Materials

The following supporting information can be downloaded at: https://www.mdpi.com/article/10.3390/foods11121691/s1, Figure S1: Calibration curve of OA in the indirect ELISA; Table S1: Parameters varied during optimization.
