# Supplementary material for: Cascade-Enhanced Lateral Flow Immunoassay for Sensitive Detection of Okadaic Acid in Seawater, Fish, and Seafood
Source: Foods. 2022 Jun 9;11(12):1691. doi: 10.3390/foods11121691 (PMC9222646; doi:10.3390/foods11121691)
Supplement: Supplementary file 1 [file foods-11-01691-s001.zip › foods-1751150-supplementary.pdf]

foods

Supplementary Material

Cascade-enhanced lateral flow immunoassay for sensitive detection of phycotoxin okadaic acid in seawater, fish, and seafood

Olga D. Hendrickson, Elena A. Zvereva, Anatoly V. Zherdev and Boris B. Dzantiev*

Bach Institute of Biochemistry, Research Center of Biotechnology of the Russian Academy of Sciences, Leninsky prospect 33, 119071, Moscow, Russia; odhendrick@gmail.com (O.H.); zverevaea@yandex.ru (E.Z.); zherdev@inbi.ras.ru (A.Z.)
* Correspondence: dzantiev@inbi.ras.ru; Tel.: +7-495-954-31-42

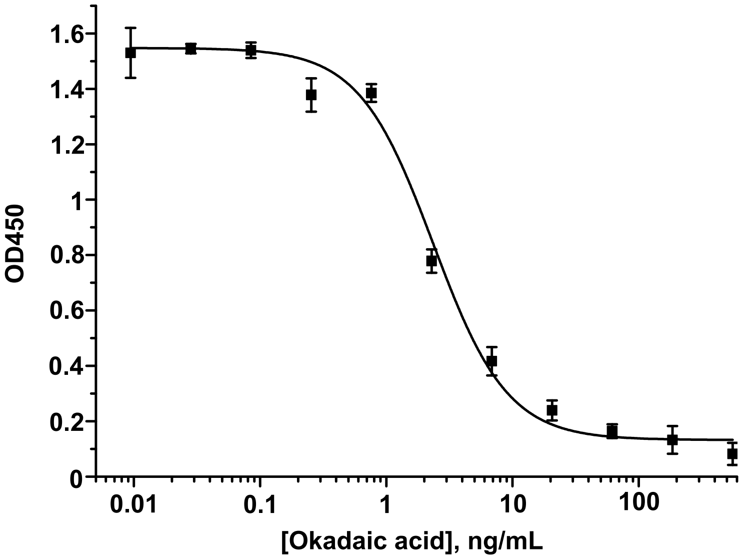

Figure S1. Calibration curve of OA in the indirect ELISA.

Table S1. Parameters varied during optimization.

Parameter	Range of varied values	Selected value
Concentration of OA–BSA in the T zone, ng/mL	0.1–0.75	0.5
Concentration of DAGI in the C zone, ng/mL	0.05–0.5	0.1
Concentration of anti-OA MAbs, μg/mL	0.02–0.2	0.1
Volume of GAMI–AuNPs, μL	2–5	2.5
Time of preincubation of the reaction mixture, min	2–5	3
Time of incubation of test strips with the reaction mixture, min	10–20	15
